# Tying the loose ends together in DNA double strand break repair with 53BP1

**DOI:** 10.1186/1747-1028-1-19

**Published:** 2006-08-31

**Authors:** Melissa M Adams, Phillip B Carpenter

**Affiliations:** 1 Department of Biochemistry and Molecular Biology, University of Texas Health Science Center, Houston, TX 77030, USA

## Abstract

To maintain genomic stability and ensure the fidelity of chromosomal transmission, cells respond to various forms of genotoxic stress, including DNA double-stranded breaks (DSBs), through the activation of DNA damage response signaling networks. In response to DSBs as induced by ionizing radiation (IR), during DNA replication, or through immunoglobulin heavy chain (IgH) rearrangements in B cells of lymphoid origin, the phosphatidyl inositol-like kinase (PIK) kinases ATM (mutated in ataxia telangiectasia), ATR (ATM and Rad3-related kinase), and the DNA-dependent protein kinase (DNA-PK) activate signaling pathways that lead to DSB repair. DSBs are repaired by either of two major, non-mutually exclusive pathways: homologous recombination (HR) that utilizes an undamaged sister chromatid template (or homologous chromosome) and non- homologous end joining (NHEJ), an error prone mechanism that processes and joins broken DNA ends through the coordinated effort of a small set of ubiquitous factors (DNA-PKcs, Ku70, Ku80, artemis, Xrcc4/DNA lig IV, and XLF/Cernunnos). The PIK kinases phosphorylate a variety of effector substrates that propagate the DNA damage signal, ultimately resulting in various biological outputs that influence cell cycle arrest, transcription, DNA repair, and apoptosis. A variety of data has revealed a critical role for p53-binding protein 1 (53BP1) in the cellular response to DSBs including various aspects of p53 function. Importantly, 53BP1 plays a major role in suppressing translocations, particularly in B and T cells. This report will review past experiments and current knowledge regarding the role of 53BP1 in the DNA damage response.

## Background

**Figure 1:**
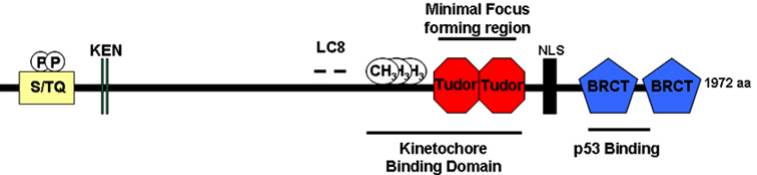
Human 53BP1 is composed of 1,972 amino acids and contains several noteworthy structural features as discussed throughout the text. p53 binds to the N-terminal BRCT motif and linker sequence of 53BP1. 53BP1 possesses numerous PIK phosphorylation sites (S/TQ) and is phosphorylated on serine residues 25 and 29 *in vivo*. Like BRCA1 and Mdc1 and the yeast Rad9 and Crb2 proteins, 53BP1 possesses two repeating C-terminal BRCT motifs. In addition, 53BP1 contains a tandem tudor domain, a stretch rich in glycine and arginine residues (1396–1403) that is methylated by the PRMT1 arginine methyltransferase in vivo and in vitro, LC8 binding sites and two potential KEN boxes (aa 54–60 and 85–91), sequences known to interact with the anaphase promoting complex (APC).

The *p53 *gene encodes a tumor suppressor whose primary function is in transcription. *p53 *is inactivated or disrupted in ≥50% of all human cancers. Mdm2, an E3 ubiquitin ligase, interacts with the N-terminus of p53 and ubiquitinates it, thus marking the protein for destruction by the proteosome. ATM phosphorylates p53 in response to DSBs, an event that prevents its Mdm2-mediated degradation and results in the stabilization and accumulation of the protein [1,2]. Using the core DNA binding domain of p53 (residues 80–320) as bait in a two hybrid screen, Fields and colleagues first identified 53BP1 in 1994 [3]. Human 53BP1 is comprised of 1,972 residues and contains important structural elements including two Breast Cancer Gene 1 (*BRCA1*) C-terminal (BRCT) repeats, tandem Tudor domains, a GAR methylation stretch, two dynein light chain (LC8) binding sites, and numerous PIK kinases and cyclin-dependent (CDK) phosphorylation sites (Fig. 1). The sequences of 53BP1 that bind p53 include the C-terminal BRCT region. *In vitro*, the tandem BRCT repeats of 53BP1 (residues 1,724–1,972) bind core p53 residues with a K_d _of 6 μM as determined by isothermal titration calorimetry [4]. First identified in BRCA1, BRCT motifs have been identified in a number of proteins that are connected to DNA damage response mechanisms. BRCT motifs have been reported to participate in various processes such as transcriptional activation and they have the capacity to serve as phospho-peptide binding modules [5,6]. Because wild-type, but not mutant p53 (i.e. R175H) binds 53BP1, the conformation of p53 appears crucial for the 53BP1-p53 interaction [3]. To date, p53 is the only factor reported to directly interact with any of the two BRCT motifs of 53BP1. Subsequent transient co-transfection experiments with 53BP1 and p53 reporter plasmids suggested that 53BP1 enhanced p53-mediated transcription [7]. Another report suggesting a link between 53BP1 and transcription came with the identification of a 98 amino acid region of murine 53BP1 (corresponding to human residues 1,179–1,277) that interacted with the p202 transcription factor [8]. The significance of this interaction remains uncertain.

The crystal structure of the recombinant BRCT motifs of 53BP1 and the central DNA binding domain of p53 (core) has been solved [9,10]. Here, p53 binds to the N-terminal BRCT motif and the linker region of 53BP1. Importantly, the structural analysis also reveals that the same p53 residues are involved in binding both 53BP1 and DNA, making it very difficult to imagine how 53BP1 could enhance p53-mediated transcription. This point has been previously discussed by Halazonetis and co-workers [11]. Although it appears very unlikely that 53BP1 enhances p53-mediated transcription as once suggested, one report has concluded that 53BP1 positively regulates the *BRCA1 *promoter [12]. In this study, the p53-proficient U20S cell line was co-transfected with siRNA molecules directed against 53BP1 and a luciferase reporter construct under the control of the minimal *BRCA1 *promoter. This resulted in >70% inhibition of promoter activity [12]. Furthermore, using chromatin immunoprecipitation (ChIP) assays, 53BP1 was shown to bind to an imperfect palindromic sequence within the *BRCA1 *promoter element. How 53BP1 regulates BRCA1 expression remains unknown, but it is possible that its BRCT repeats possess transactivation potential.

## Implicating 53BP1 in the biology of p53 function

### 53BP1/p53 interactions at stalled replication forks

In addition to the initial binding studies between 53BP1 and p53, some recent evidence implicates 53BP1 in the function of p53. Small interfering RNA (siRNA) knockdown of 53BP1 in U20S cells revealed a delayed accumulation of p53 in response to IR, an inducer of DSBs [13]. However, *53BP1*-defective T cells, as generated from knock-out mice (*53BP1*^-/- ^; see below), failed to show altered levels of p53 accumulation in response to IR [14], indicating possible differences in cell type and species. Additionally, the expression of the two p53 target genes, *Mdm2 *and *p21*, was normal in *53BP1*^-/- ^thymocytes [14], further illustrating the unliklihood that 53BP1 acts as a transcriptional co-activator of p53. However, in a pathway dependent upon activation of Chk1, 53BP1 was shown to regulate the accumulation of the ReqQ helicase BLM and p53 to sites of stalled replication forks [15]. In addition to the well described role of p53 as a transcription factor, the protein has also been shown to mediate recombination events through its recruitment to stalled replication forks and other abnormal DNA structures (i.e. Holliday junctions) in a manner dependent upon BLM [15]. Thus, 53BP1 is required for the localization of p53 to stalled replication forks.

### 53BP1/p53 and dynein motors

Another biochemical link between 53BP1 and p53 has been uncovered in experiments designed to understand the dynein microtubule motor complex [16]. Previous studies demonstrated that p53 undergoes cytoplasmic dynein-dependent nuclear translocation in response to DNA damage. Because p53 is not known to bind directly to the dynein motor complex, Zhang and colleagues hypothesized that adaptor proteins would tether p53 to the minus-end directed microtubule motor complex [16]. To identify such factors, a two-hybrid screen using the dynein 8 kD light chain protein (LC8) as bait was performed. Here the investigators found that LC8 robustly binds 53BP1 *in vitro*. In fact, 53BP1 contains two tandem LC8-binding motifs (Fig. 1). Thus, 53BP1 has been proposed to link p53 to the microtubule motor complex through its dynein binding capacity. How this relates to the previously described localization of 53BP1 to the kinetochore [17,18] is not clear.

### Influence of 53BP1 on Mdm2/p53 signaling

Because roughly one half of all tumors have wild-type p53 activity, one approach for activating and stabilizing p53 in tumor cells could involve disrupting the Mdm2-p53 interaction. This would be expected to induce a cell cycle arrest in apoptosis-resistant tumor cells. In this regard, p53 function is a key target for therapeutic intervention. The small molecule nutlin-3 has been shown to abrogate the Mdm2-p53 interaction leading to p53 stabilization [19]. Recently, a loss-of-function genetic screen designed to identify components of the p53 network that mediate the cytotoxic effects of nutlin-3 in human tumor cells identified 53BP1 [20]. Here, an siRNA bar code screen using the p53 proficient MCF-7 cell line identified *53BP1 *as an essential gene for the drug-induced phenotype, a result that provides further evidence for a connection between 53BP1 and p53. Thus, 53BP1 mediates the antiproliferative effects of nutlin-3, a finding the authors suggest indicates that intrinsic DNA damage signaling in MCF-7 cells (and hence 53BP1 activity) is required for nutlin-3 cytotoxicity [20]. This provides strong evidence for a role for 53BP1 in the biology of p53 and Mdm2 in MCF-7 cells. These authors also found that the deubiquitanase USP28 also participated in nutlin-mediated cytotoxicity. USP28 has been recently been shown to regulate the Chk2-p53-PUMA pathway and this is particularly of interest as USP28 has been identified as a 53BP1-binding protein ([21]; Table 1). These two studies clearly show that 53BP1 functions in the biology of p53. Although USP28 has been proposed to use 53BP1 as a substrate, ubiquitylated 53BP1 has not been detected [21].

**Table 1:** Summary of proteins known to interact with 53BP1.

**Protein**	**Comments**	**References**
p53	53BP1 was discovered as a protein that binds to the central core DNA binding domain of p53 in a yeast two-hybrid assay.	[3]
RIF1	53BP1 is required for IRIF formation of RIF1, a human homolog of a yeast telomeric protein that participates in the intra S-phase checkpoint.	[104]
RPA2	53BP1 co-immunoprecipitates with RPA2, single-stranded DNA binding complex. Association is IR dependent.	[105]
Jab1	Jab1 was shown to bind to 53BP1 in a yeas two-hybrid screen. Jab1 may be responsible for the observed hyperphosphorylation of 53BP1 in M phase in response to nocodazole.	[93]
Dynein LC8	53BP1 possesses two tandem LC8 binding sites (Fig. 1) and may bind to the dynein motor complex protein in a manner that regulates p53 function.	[16]
BRCA1	53BP1 is required for optimal IR-inducible phosphorylation of BRCA1 at S1472; 53BP1 is part of the "BASC" complex and interacts with other components of this complex.	[13, 106]
Chk2	53BP1 is possibly required for optimal Chk2 phosphorylation at T68 in response to IR.	[13, 29]
H2AX	A fragment of 53BP1 containing residues 956–1354 were reported to bind phosphorylated peptides corresponding to the C-terminal tail of γ-H2AX.	[107]
Mdc1	53BP1 binds to the BRCT repeat protein Mdc1. 53BP1 IRIF formation is dependent upon functional Mdc1.	[38–40, 50]
Smn1	53BP1 co-localizes with and co-immunoprecipitates with Smn1, an Artemis-like protein.	[108]
Artemis	53BP1-Artemis interactions were reported from cells transfected with 53BP1 expression vectors	[88]
ATM	53BP1 has been reported to co-immunoprecipitate with ATM. 53BP1 is clearly an ATM substrate, but may also participate upstream of ATM activation	[11, 23, 26, 28, 58]
Histones H3 and H4	The tandem Tudor domains of 53BP1 have been reported to bind various methylated states of histones H3 and H4 as discussed in the text.	[58, 60]
Rad51	Although 53BP1 does not appear to function in break-induced IR, it has been detected to co-localize to I-SceI induced DSBs.	[52, 53]
HDAC4	53BP1 and HDAC4 bind together and co-localize to IRIF. The stability of 53BP1 depends on HDAC4 and vice versa.	[18]
PTIP	PTIP shows increased association with 53BP1 in response to IR and in an ATM-dependent manner. Amino acids 1052–1710 of 53BP1 are necessary for interaction with PTIP.	[41]
USP28	Deubiquitinase that binds to 53BP1 and controls Chk2-p53-PUMA pathway. Also, mediates cytotoxic effects of nutlin, a molecule that influences p53 stability.	[20, 21]

53BP1 interacts with various factors implicated in maintaining genomic stability through their control of DSB repair, cell cycle checkpoints, and chromatin structure. Partial summary of proteins, listed in no particular order, reported to interact with 53BP1 throughout various in vitro and in vivo techniques including yeast-two hybrid assays and co-immunoprecipitation. See text for more details.

## 53BP1: focusing on mediating the DNA damage response through ATM signaling

In response to IR, the histone variant H2AX is rapidly phosphorylated by PIK kinases and relocalizes to sites of DNA damage [22,23]. The accumulation of phosphorylated histone H2AX (γ-H2AX) at and near sites of DSBs can be detected cytologically as irradiation induced foci (IRIF) [22,23]. Using antibodies against human and *Xenopus *53BP1, several labs found that 53BP1 forms IRIF in response to genotoxic stress, particularly agents that induce DSBs [24-27]. Such foci overlap with those previously described for H2AX and Mre11. Moreover, 53BP1 possesses numerous PIK kinase phosphorylation sites (S/TQ) and was shown to be a substrate for ATM and probably its related kinases [24,25,27]. 53BP1 phosphorylation, however, is not required for IRIF formation [28], although it clearly represents an activating event. Based upon these observations, 53BP1 was proposed to operate in DSB repair. Indeed, 53BP1 has been shown to physically associate with a number of proteins involved in various aspects of DSB repair (Table 1). A further glance into the functional role of 53BP1 in DNA damage signaling came with the analysis of mammalian cell lines depleted in 53BP1 expression through siRNA or from knock-out mice (see below) [13,29,30]. These cells revealed that 53BP1 was required for the optimal, DNA damage-inducible phosphorylation of ATM substrates such as Smc1, Chk2, and BRCA1 [13,29,30]. Furthermore, *53BP1*-deficient cells have been reported to have a mild intra-S phase (RDS phenotype) and a nearly three-fold defect in the G_2_/M checkpoint, but only at low doses of IR [13,29]. One study showed that *53BP1*^-/- ^cells had a prolonged G_2 _phase, a phenotype also reported in ATM-deficient cells [30]. This suggested that 53BP1 mediated the DNA damage response by facilitating ATM activity towards at least a subset of its substrates and raised the possibility that 53BP1 could function in ATM activation, an event that is likely to be tightly coupled to chromatin structure and function [11,29].

How cells sense and respond to DSBs has been the subject of intense research by numerous laboratories. Because the DNA repair apparatus uses damaged DNA as a substrate, the DNA damage response must be tightly linked to chromatin structure and function. In response to DSBs, the Mre11/Rad50/Nbs1 (MRN) complex is rapidly recruited to sites of DNA breaks [31,32]. In budding yeast, the Mre11 complex facilitates nucleosomal displacement near the DSB and H2A phosphorylation [33]. Here, loss of H2B and H3 also depends on the Ino80 chromatin remodeling complex [33]. In fact, ChIP analysis reveals that phosphorylated H2A in budding yeast is not associated with the break per se, but rather the Mre11 complex appears to localize to sites of DNA damage [34]. This is believed to contribute, at least in part, to ATM activation [35,36]. In addition, autophosphorylation of ATM, particularly at S1981 (S1981-P), has been linked to its activation where it occurs in response to DNA damage and changes in chromatin structure [37]. However, the role of S1981 phosphorylation is not clear because mice deficient in the mutant appear largely normal for several ATM functions, including those that occur in B and T cells [38]. ATM activation, as measured by S1981 autophosphorylation, also occurs in response to changes in chromatin structure in the apparent absence of DNA breaks [37]. Moreover, the MRN complex is also believed to contribute to the activation of ATM kinase [35,36]. Acetylation of ATM by Tip60 has also been attributed to its activation [39]. Thus, ATM activation appears to be a highly regulated event where autophosphorylation at S1981 may represent just one facet of its activation.

ATM phosphorylates and contributes to the activation of a number of important DNA damage response regulators. In response to IR, ATM phosphorylates 53BP1 on serine residues 25 and 29 [28]. Importantly, ATM phosphorylates the C-terminal tail of H2AX, generating γ-H2AX which directly recruits and binds Mdc1 (formerly known as NFBD1), a factor that, like 53BP1, contains two C-terminal BRCT repeats in addition to a forkhead domain [40-42]. Through the BRCT motifs of Mdc1, the γ-H2AX-Mdc1 complex recruits another ATM molecule, thus amplifying the original DNA damage response signal through the creation of a positive feedback loop [40-44]. As a consequence, neighboring H2AX molecules that reside in a subset of histone octamers peripheral to the DNA break become phosphorylated, resulting in the generation of γ-H2AX molecules that lie up to one megabase from the actual site of the DNA break.

A variety of proteins, including 53BP1, have been shown to localize to IRIF in many cell types. In lymphoid B cells, 53BP1 localizes to the switch locus of murine chromosome 12 [14,45]. The precise biological role of IRIF is unknown, but they are in all probability associated with DNA repair. Indeed, the accumulation of H2AX to IRIF is routinely used as a marker for DSBs. Because many cancer cells and tissues exhibit constitutive 53BP1 foci, activation of DNA damage response pathways may be an early feature in human tumorigenesis, probably as a consequence of the intrinsic genomic instability in these cells [46,47]. This could provide a selection for p53 loss during tumorigenesis and has been proposed to explain the large frequency of *p53 *mutagenesis in cancer [47]. Indeed, loss of 53BP1 function correlates with cancer progression in human tumors [46,47]. How widespread 53BP1 dysfunction is in human tumors has not been thoroughly addressed. Interestingly, a human patient with a myeloproliferative disorder was found to have a translocation that generated a fusion protein between 53BP1 and the platelet-derived growth factor (PDGF; [48]). Because, in this scenario, PDGF tyrosine kinase activity depends on the ability of its fusion partner sequences to form oligomers, it was proposed that 53BP1 could form oligomers [48]. Indeed, 53BP1 oligomerizes *in vivo*, but the significance of this remains unknown [49].

Although some siRNA studies concluded that 53BP1 and Mdc1 IRIF formation occurs independently of each other [40,50,51], it is now clear that both γ-H2AX and Mdc1 are required for the accumulation of 53BP1 to IRIF [41,42,52]. In addition, the histone acetyltransferase (HAT) co-factor Trrap and HAT Tip60 complex (Trrap-Tip60) is required for 53BP1 IRIF formation [53]. Trrap-Tip60 binds to chromatin near DSBs and Trrap-deficient cells are impaired in break-induced HR [53], underscoring the intimate link between DNA repair and chromatin structure (see below). Since Trrap is required for 53BP1 IRIF formation and influences HR [53], does this necessarily mean that 53BP1 functions in break-induced HR? Apparently not, as 53BP1 plays no role in this process when tested with in an I-*Sce *I-based system developed in the Jasin laboratory that measures gene conversion [54]. Despite this, ChIP experiments indicate that 53BP1 localizes with the Rad51 paralogs Rad51A and Rad51C to I-*Sce*I-induced DSB foci [55]. Moreover, in the absence of extrinsic DNA damage, 53BP1 localizes to "nuclear dots" during S/G_2 _[53], a phenomenon similarily reported for Rad51 and BRCA1 [56]. Unlike 53BP1, H2AX and Mdc1 have been reported to participate in HR [54,57,58], further illustrating the differences between regulators of DSB repair pathways. *53BP1*^-/- ^fibroblasts, however, display a hyper-rec phenotype, suggesting that the protein suppresses aberrant, spontaneous recombination (as opposed to break-induced HR). Clearly, 53BP1 does not act redundantly with H2AX and Mdc1 during the DNA damage response.

## Linking 53BP1 function with chromatin structure

Although unlikely to be a structural component of chromatin, 53BP1 possesses several intriguing links to the biology of chromatin, particularly with respect to the regulation of histone modifications [59,60]. IR-inducible phosphorylation of 53BP1, which likely represents the "active" protein, associates with chromatin [61]. In terms of CSR in B cells, one possibility is that 53BP1 synaptic function provides a scaffold for coordinating DNA repair through modulating chromatin structure in the language of a histone code. First, in addition to the well documented role of γ-H2AX-dependent recruitment of 53BP1 to IRIF, 53BP1 possesses tandem Tudor motifs (53BP1^TT^) [59,60] which have been reported to associate with various methylated lysine residues in histones H3 and H4 [60,62]. This includes lysines K4, K9, and K20 in histone H4 and H3 K79 (by the Dot1 methyltransferase). Intriguingly, in fission yeast, methylation of histone H4 at K20 has been linked to DNA repair through its interaction with Crb2 [63], a potential 53BP1 orthologue (see below). No such function for H4 K20 has been reported in mammalian cells. Tudor motifs resemble chromodomains at the structural level and associate with methylated arginine and lysine residues [64]. For example, 53BP1^TT^, the minimal region of the protein sufficient for IRIF formation, binds to H3 K79-CH_3 _independently of DNA damage [60]. Although this modification is constitutive, lysine 79 is sterically inaccessible under normal conditions as it lies in the nucleosome core. Because of this, 53BP1 has been proposed to sense changes in chromatin structure that occur "upstream" of the DNA break [60]. In this regard, a perturbation in chromatin structure, as introduced through a nearby DSB, is thought to expose H3-K79-CH_3 _and allow for 53BP1 binding and execution of its essential functions in DSB repair. Indeed, 53BP1 apparently binds H3-K79-CH_3 _under conditions that perturbed chromatin structure without introducing DSBs [60]. In the case of isotype switching in activated B cells, 53BP1 activation could be induced through transcriptional events that alter chromatin structure prior to the genesis of AID-dependent DNA breaks. Thus, recruitment of 53BP1 to chromatin during DSB repair may proceed through multiple histone modifications and is likely connected to its role in a non-classical form of end joining ("anchoring") [65].

As it operates early in the DNA damage response and associates with modified histones, 53BP1 is a strong candidate for participating in both DSB repair and chromatin remodeling in the vicinity of the break or in response to other types of chromatin perturbation. In this manner, DNA repair by 53BP1 is coordinated with IRIF factors like H2AX and Mdc1 in conjunction with its ability to influence chromatin architecture. Previous studies have indicated that in response to DNA damage yeast H2A recruits chromatin remodeling complexes [66-68]. Additional, non-mutually exclusive mechanisms may also be operative in this process. For example, the PRMT1-dependent methylation of 53BP1 at arginine residues [49,69] within its GAR motif (Fig. 1) may form an epigenetic signal that participates in 53BP1 function. However, GAR methylation does not influence DNA binding or IRIF formation by 53BP1 [49]. Furthermore, 53BP1 protein levels are reciprocally regulated by the histone deacetylase 4 (HDAC4; [68]). Because one might anticipate that chromatin near a DSB would exist in a "relaxed" form that permits the DNA repair apparatus access to the sites of damage [70], perhaps 53BP1 activity influences acetylation levels through HDAC4 function.

## 53BP1 and DNA end joining: lessons from lymphocytes

### 53BP1 mouse models

Key functional insight into the role of 53BP1 in DSB repair has been gained from studying murine T and B cells in *53BP1*-defective (*53BP1*^-/-^) mice. The first two descriptions of *53BP1*^-/- ^animals were reported from the Chen and Carpenter labs [30,71]. Both animals appear to be null mutations and possess very similar, but not identical, phenotypes. A third *53BP1*^-/- ^mouse model has been reported but has not been thoroughly characterized [72]. In one mouse model, 53BP1 has been reported to function as a haploinsufficient tumor suppressor [14]. Previous studies with *H2AX/p53 *double mutant animals, which develop T and B lineage tumors, also showed a haploinsufficient phenotype for *H2AX *in tumor suppression [73,74]. Because the H2AX variant resides in up to 30% of histone octamers, haploinsufficiency was argued to reflect a role of the protein as a structural component of chromatin [73,74]. Interestingly, *53BP1*^-/- ^mice resemble, in some important ways, *H2AX*^-/- ^and *ATM*^-/- ^mice as well as the recently described *Mdc1*^-/- ^mice (Table 2[41,75-77]). Like *H2AX*^-/- ^and *ATM*^-/- ^mice, *53BP1*^-/- ^mice are growth retarded, sensitive to IR, and immune deficient [41,75-77]. In contrast to *ATM*^-/- ^mice which die of T cell lymphomas with clonal translocations, *53BP1*^-/- ^and *H2AX*^-/- ^mice are mildly tumor prone [30,71]. *53BP1*^-/- ^animals are fertile and have reduced thymus cellularity [30,71]. Lack of 53BP1 function results in mice that are immune deficient as they are severely impaired in performing the B cell-specifc process of IgH class switch recombination (CSR or isotype switching) [54,78].

**Table 2:** Tumor susceptibility in mice defective in *53BP1 *and related DNA damage response genes.

**Defective Gene(s)**	**B cell lymphoma**	**T cell lymphoma**	**References**
*53BP1*	None	Low-mild	[29, 69]
*p53*	None	All aneuploid with no clonal breaks	[109–111]
*53BP1/p53*	t(12;15) translocations that amplify *c-myc*	Aneuploidy and clonal translocations that include TCRα locus	[14, 85]
*H2AX*	None	Low-mild	[73, 74]
*H2AX/p53*	t(12;15) translocations that amplify *c-myc*	75% of animals clonal translocations; *H2AX*^+/-^*p53*^-/- ^animals are aneuploid or possess clonal translocations	[71, 72]
*NHEJ*	None	None	[77]
*NHEJ/p53*	Pro-B cell lymphoma	None	[77, 89–92]
*Mdc1*	None	Low	[39]
*ATM*	None	100% of animals with clonal translocations at TCR locus of chromosome 14	[75]

Phenotypes of *53BP1 *-deficient murine animals relative to those of its related DSB repair factors with particular emphasis on tumor susceptibility. Murine animals defective in 53BP1 function share overlapping features to *H2AX*, *Mdc1 *and *ATM *-deficient animals in that they are growth retarded, sensitive to IR and immune deficient as they are impaired in isotype switching. Like H2AX and any of several components of the NHEJ apparatus, *53BP1*^-/- ^animals are relatively non-tumor prone unless combined with *p53 *deficiency. See text for details.

### DNA repair in immune development

**Figure 2:**
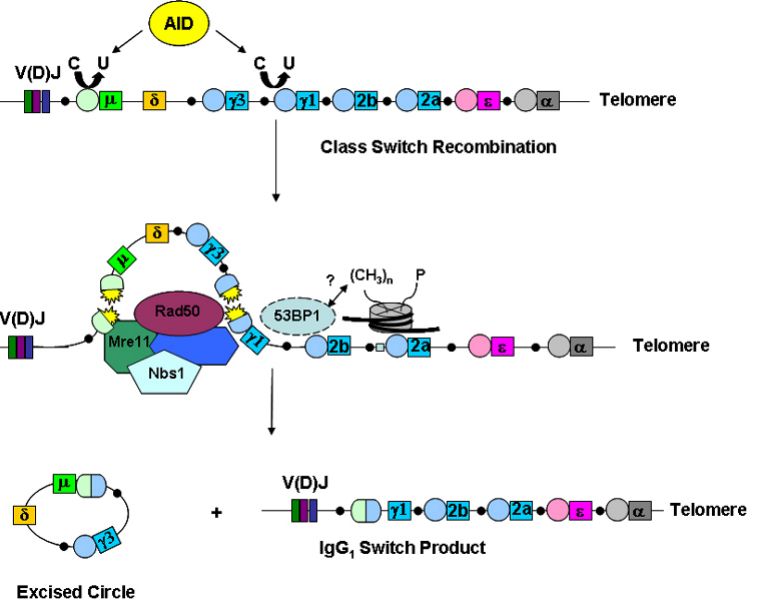
Class-switch recombination is a NHEJ event involving the exchange of heavy chain constant regions with the previously assembled V(D)J exons. AID induces lesions at the switch regions (circles) upstream of a given IgH gene which are converted to DSBs. These DSBs recruit various factors, including Mre11/Nbs1/Rad50, that are important for the resolution of the break. The end result of CSR is the replacement of the mu exon with another constant region gene (IgG_1 _shown). 53BP1 performs an anchoring role at the IgH locus during this process that might involve interactions with nucleosomes near the break through various histone modifications (see text for details). This is thought to modulate chromatin in this area to facilitate DNA repair.

During the development of the immune system, both T and B cells use NHEJ to assemble the exons encoding immunoglobulins (Igs) and T cell receptors (TCR) by V(D)J recombination [79,80]. Additionally, mature B cells may express different Ig constant regions through the process of CSR, a DSB-mediated, end-joining event that requires successful completion of V(D)J recombination, germline transcription through the switch (S) locus on murine chromosome 12, and cell proliferation (Fig. 2) [79]. CSR is an inducible event that occurs at a defined (and very important) chromosomal location and therefore represents a unique system for examining DNA repair and related processes in mammalian and lymphoid cells. Although V(D)J recombination and CSR are both DSB repair events that are completed by any of several ubiquitous NHEJ proteins, they require a different set of overall factors (Fig. 2) [79,81-84]. For example, DSBs in V(D)J recombination are initiated by the RAG endonuclease, but CSR is initiated by the activation induced deaminase (AID) [79,81-84]. By deaminating cytosine to uracil, AID activity induces DNA repair pathways that result in the formation of DSBs throughout the switch locus, as the uracil residues are thought to be processed into DSB intermediates [79,81-84]. During CSR, multiple AID-derived DSBs may occur within the S region, leading to both internal deletions and those repair events that properly join S region DNA fragments leading to productive isotype switching. Because S regions that lie up to approximately 100 kB away from each other they are likely to be brought into close physical proximity during synapsis to ensure a productive recombination events and successful isotype switching. Chromosomal translocations involving the immunoglobulin switch region are feature of B cell malignancies and therefore understanding how DNA repair factors like 53BP1 function will provide important insight into the mechanisms of translocation-induced tumorigenesis.

### Defective DNA repair in 53BP1^-/- ^B cells reveals a major role in the end joining process of CSR

Through various measurements, 53BP1 appears largely, but probably not exclusively, dispensable for V(D)J recombination at T cell receptor (TCR) and IgH loci [54,78]. 53BP1, however, plays a major role in the B cell-specific process of CSR [54,78]. DNA repair at IgH during CSR represents a specialized form of DSB repair that proceeds through H2AX and 53BP1-mediated pathways [54,75,76,78,80], CSR depends on S region transcription and recombination is targeted to individual S regions from transcription that occurs from intronic promoters [79,82]. Through coordinated synapses over long ranges, CSR serves as a regulated DNA repair event in B cells that operates in close association with S region transcription and AID activity. It is now very clear that secondary isotype formation through CSR is severely reduced in *53BP1*^-/- ^B cells [54,78]. In fact, this DNA repair defect is more severe than those deficiencies observed for ATM, H2AX, and Mdc1 defective B cells [82,84]. In those rare cases where *53BP1*^-/- ^B cells did successfully perform isotype switching, the sequence junctions at S_μ_-S_γ1 _appeared normal, indicating that defects in 53BP1 function do not influence resolution of the repair event [78]. Moreover, since *53BP1*^-/- ^B cells proliferate normally, are competent for S region transcription, and have wild-type levels of AID expression, the CSR defect in these cells was clearly not due to the initiation or resolution of the DNA break. The DSB defect was therefore proposed to occur at the level of synapsis, or DNA end joining [78]. Consistent with this, a PCR strategy that amplifies DNA only after productive CSR events have taken place showed that the defect in *53BP1*^-/- ^B cells occurred at the level of recombination [78]. Thus, in B cells, 53BP1 participates in DNA end joining during CSR probably through an end joining-like function. Because the RAG complex possesses synaptic activity, this could obviate or reduce the need for 53BP1 function during V(D)J recombination. These results regarding a synaptic function for 53BP1 were interesting in light of a prior report that showed that a recombinant fragment of human 53BP1 encompassing residues within the kinetochore binding domain (Fig. 1) stimulated dsDNA end joining activity by XRCC4/LigIV, but not T4 ligase *in vitro *[61]. A more recent study using the chicken B cell line DT40 has confirmed a major role for 53BP1 in end joining [85]. In addition, previous reports suggested that H2AX tethers broken chromosome ends through long range synapsis at S regions [45,73,74] leading to the proposal that H2AX participates in an end joining process distinct from classical NHEJ, termed "anchoring" [86]. Similarly, 53BP1 has been proposed to operate in a non-classical, or alternate form of end joining [65,78]. In this capacity, H2AX and 53BP1 facilitate DNA repair of broken chromosomes by anchoring DNA ends in preparation for end processing and ligation by the NHEJ apparatus, an activity that can suppress translocations.

To further illustrate a role for 53BP1 in DNA end joining, a two-color FISH assay using probes designed to hybridize to sequences external to the V(D)J and CSR regions was used [87]. Under wild-type conditions where CSR is normal and DNA repair is intact, two distinct, essentially non-overlapping FISH signals (red and green) are readily seen at the *Igh *locus in metaphase spreads [87]. In contrast, a failure to repair DSBs induced at IgH would result in "split signals" as the telomeric-proximal FISH signal would be separated from the remaining chromosome. In the case of *53BP1*^-/- ^B cells, a significant number of "split" signals as seen by single red and green spots was observed [87]. The number of split signals was dependent on AID function [87], indicating that in the absence of 53BP1 function, AID-induced DSBs generate chromosomal breaks refractory to repair [87,88]. Similar conclusions were reached from experiments using *H2AX*^-/-^, *Mdc1*^-/-^, and *ATM*^-/- ^B cells [87,88]. Some 53BP1 breaks progressed into chromosome 12-derived translocations (i.e. *Igh-c-myc*) [87,88]. An increase in chromosome 12 dicentrics was also observed (CSR often occurs on both alleles). In conclusion, 53BP1 deficiency results in the inability to "anchor" chromosomal breaks and creates a cellular context permissive for the genesis of translocations.

**Figure 3:**
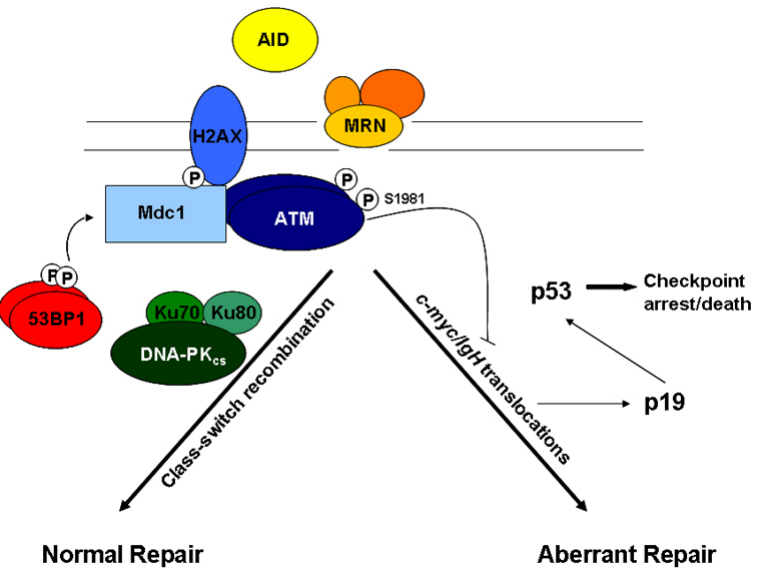
During class-switch recombination, AID-induced breaks are repaired by the DSB repair proteins such as Mre11/Nbs1/Rad50, H2AX, Mdc1, 53BP1, and components of the NHEJ apparatus (Ku70, Ku 80, DNA-PK). Failure to perfom DSB repair activates ATM, leading to p53 stabilization. Alternatively, breaks are resolved by translocations often involving *c-myc*. These translocations are suppressed by ATM or by activation of the p19-dependent pathway leading to the activation of p53, resulting in checkpoint arrest and cell death. Adapted from reference 88 with permission.

53BP1, H2AX, Mdc1, and ATM have similar phenotypes and physically interact. Therefore, these factors likely function in a common biochemical pathway that suppresses translocations, particularly in cells of lymphoid origin. In the absence of 53BP1 activity, the failure to "anchor" or perform non-classical, alternate end joining function triggers an ATM-dependent signaling pathway that activates p53 for cell cycle arrest (and/or apoptosis) (Fig. 3) [87,88]. In addition, AID-dependent, unrepaired DSBs may also be repaired through translocations (particularly to *c-myc*), which are suppressed by ATM and p19 signaling pathways [88]. Interestingly, the majority of genomic instability in *53BP1*^-/- ^B cells occurs at the IgH locus suggesting that this factor performs a very specific role in CSR. One striking feature of *53BP1 *deficiency in fibroblasts, T cells, and B cells is the apparent lack of genomic instability in unchallenged cells [71,87,89]. When *53BP1*^-/- ^B, T, or fibroblast cells are treated with IR or during DNA repair in lymphoid cells, increases in breaks, gaps, and translocations become apparent [87,89]. This feature of 53BP1 distinguishes it from *H2AX, Mdc1*, and *ATM *deficiencies which are characterized by more severe forms of genomic instability in resting cells [41,87,88]. Despite this, the CSR defects in *H2AX*^-/- ^and *ATM*^-/- ^genetic backgrounds are moderate compared to the severe phenotype observed in 53BP1 deficiency. Why *53BP1*^-/- ^B cells have profound defects in CSR is currently unknown, but it could be related to the nature of the repair process itself. For example, one study has suggested that focus forming proteins like 53BP1 collaborate with ATM and Artemis to resolve complex DNA end structures (i.e. those that cannot be directly ligated) through end joining [90]. Although, its role in V(D)J recombination has been well described [79,80], Artemis is not required for CSR [91]. Such difficult DNA repair substrates might be expected to occur for a small subset of DSBs and this was proposed to explain the reasons underlying the radiosensitivity in Artemis-defective fibroblasts, although defects in cell cycle checkpoints may also be an important determinant of this [92]. If CSR at the IgH locus can be considered a complex form of DSB repair due to the unusual method for generating breaks by AID and because of the long-range synapses generated during repair, this could undelie the strong phenotype of 53BP1 in CSR.

### 53BP1 and p53 synergize in tumor suppression

Murine animals defective in *53BP1*, *H2AX*, *Mdc1*, and NHEJ components are relatively non-tumor prone, a result that is probably due to intact p53 surveillance mechanisms (Table 2). Mice defective in *p53 *have been well described: at an average age of 5 months, 75% of the animals die of T cell lymphomas characterized by their aneuploid nature with little signs of clonal DNA translocations [73,74]. B cell lymphomas are very rare in *p53 *nullizygous mice. However, when combined with mutations in *p53, NHEJ/p53, H2AX/p53*, and *53BP1/p53 *double-mutant animals rapidly die of T and/or B cell lymphomas [14,73,74,89,93,94]. Tumorigenesis occurs as early as 7 weeks under these conditions and has been demonstrated to be mechanistically distinct from p53 due to its altered timing and change in tumor spectrum [73,74,89,93,94]. *NHEJ/p53 *animals rapidly develop pro-B cell lymphomas due to failure to complete V(D)J-mediated end joining [79,93,94]. This DNA repair defect results in translocation of IgH with *c-myc *(*N-myc *in the case of artemis; [91]) and the resulting amplification of *c-myc *through breakage fusion bridge cycles [93,94]. Like *NHJE/p53 *animals, *53BP1/p53 *mutants develop, albeit at a reduced frequency, pro-B lymphomas with clonal translocations between IgH and *c-myc *[14]. Because pro-B cell lymphomas (IgM^-^) are indicative of a failure to successfully complete V(D)J recombination, this suggests that 53BP1 participates in some aspect of this process. In support of this, some *53BP1/p53 *T lineage tumors harbor clonal translocations at the TCRα locus [14]. Although V(D)J defects in *53BP1*^-/- ^cells have not been detected by standard measurements [54,78], subtle defects below the levels of assay detection may be selected for during T cell expansion.

The remainder of *53BP1/p53 *T cell lymphomas possessed clonal translocations in chromosomes that do not possess antigen receptor loci or were aneuploidy with little signs of DNA breaks [54,78]. With respect to the latter, 53BP1 tumor suppression resembles p53-driven aneuploidy, a form of genomic instability linked to centrosome hyperamplification. Despite this, *53BP1*^-/- ^cells have normal centrosome numbers [14]. Thus, 53BP1 appears to suppress tumorigenesis by two distinct mechanisms: DSB repair and aneuploidy (the latter of which occurs in a manner distinct from p53-driven aneuploidy). How p53 suppresses aneuploidy remains an open question, although some possible clues have emerged. In addition to the cell cycle checkpoint defects reported for *53BP1*^-/- ^cells [13,28,29], 53BP1 localizes to the kinetochore and attaches to chromosomes improperly aligned at the metaphase plate [17]. Despite this, *53BP1*-defective cells have no apparent role in the mitotic spindle checkpoint [14]. Moreover, 53BP1 is hyperphosphorylated in M phase in response to nocodazole [17], and this has been partially attributed to the JNK kinase [95]. 53BP1 has also been reported to associate with the anaphase promoting complex subunit Cdc27 [96]. Deciphering the role of 53BP1 in suppressing aneuploidy promises to lead to exciting connections between DSB repair, cell cycle checkpoints, and lymphomagenesis.

## DNA damage signaling in yeast: learning from potential 53BP1 orthologues

On first glance, there appear to be no obvious orthologues of 53BP1 in yeast. However, Rad9 and Crb2 in budding and fission yeast, respectively, possess similar functional elements (Tudor and BRCT) and parallel properties with 53BP1, despite low overall sequence homologies. Therefore, DNA damage response mechanisms in yeast will be instructive for understanding mammalian 53BP1 function. For example, Rad9 is phosphorylated in response to DNA damage by yeast PIK kinases [97]. Interestingly, loss of Dot1 (and therefore methylation of H3-K79) prevents activation of Rad9 and decreases its localization to sites of DSBs [98]. Inhibition of IR-dependent phosphorylation of H2A yields cells with very minor checkpoint defects [63,99,100]. Despite some interesting parallels between 53BP1 and Crb2, there are some distinct differences. For example, the BRCT motifs of Crb2 are required for both homo-oligomerization and IRIF formation [101], but the sequences sufficient for this in 53BP1 lie upstream of the GAR motif and in the Tudor domains, respectively (Fig. 1).

As in higher eukaryotes, phophorylation of yeast histone 2A (γ-H2A), occurs rapidly in response to DSBs, indicating that this event is highly conserved. The yeasts, however, do not possess orthologues to Mdc1 and BRCA1. Yeasts defective in H2A phosphorylation have, at best, mild defects in NHEJ and checkpoint signaling [63,99]. Rad9, Crb2, and 53BP1 (at least at low doses of IR) are required for cell cycle arrest in response to DNA damage [13,28,102]. In fission yeast γ-H2A recruits Crb2 to sites of DNA damage [102]. Interestingly, the accumulation of Crb2 at IRIF in fission yeast is dependent on both γ-H2A and histone H4 methylation at K20 [63,103], a modification that plays a role in DNA repair in fission yeast [63]. Crb2 IRIF formation appears independent of Dot1 activity as this methyltransferase does not appear to exist in the fission yeast genome [103]. Furthermore, in the absence of both H2A and H4 K20 histone modifications, Crb2 activity remains largely functional [103]. Here an alternate recruitment pathway appears sufficient for IRIF formation and checkpoint activation. Such a pathway requires CDK phosphorylation of Crb2 at T215. Crb2 phosphorylation at T215 phosphorylation is important for its association with the BRCT protein Cut5, an orthologue of mammalian TopBP1 [100]. Moreover, hypomorphic *crb2-T215A *cells have checkpoint maintenance defects [100,102]. Therefore, multiple DNA damage signals are programmed into Crb2 function, allowing it to coordinate DNA repair with cell cycle progression and chromatin remodeling.

## Summary

53BP1 plays an important role in DNA damage signaling pathways through its ability to influence the function of a variety of factors including ATM and p53 (Fig. 3). Importantly, 53BP1 participates in DNA repair through its role as a tumor suppressor protein, particularly in cells of B and T origin. Here, 53BP1 collaborates with H2AX and Mdc1 to participate in a novel form of end joining termed "anchoring". In this setting, 53BP1 is recruited to or near sites of DSBs where it coordinates DNA repair in the context of chromatin structure, most likely through the language of a histone code. Whether 53BP1 participates in chromatin remodeling events remains an open question. Given its large size, multiple domains and various interacting partners, 53BP1 is well poised to operate as a mediator in multiple DNA damage signaling events.
